# Comparison of Analgesic Efficacy of Ultrasound-Guided Interscalene Block Versus Continuous Subacromial Infusion for Postoperative Analgesia Following Arthroscopic Rotator Cuff Repair Surgeries: A Randomized Trial

**DOI:** 10.7759/cureus.13500

**Published:** 2021-02-22

**Authors:** Suman Saini, Anju Gupta, Shruti Mahesh Rao, Bhavya Krishna, Saveena Raheja, Rajeev Kumar Malhotra, Dr Nishkarsh Gupta

**Affiliations:** 1 Anesthesiology, Vardhman Mahavir Medical College and Safdarjung Hospital, Delhi, IND; 2 Anesthesiology, Pain Medicine and Critical Care, All India Institute of Medical Sciences, Delhi, IND; 3 Anesthesia and Critical Care, Vardhman Mahavir Medical College and Safdarjung Hospital, Delhi, IND; 4 Delhi Cancer Registry, All India Institute of Medical Sciences, Delhi, IND; 5 Onco-Anesthesiology and Palliative Medicine, All India Institute of Medical Sciences, Delhi, IND

**Keywords:** brachial plexus block, pain, postoperative, shoulder, surgery

## Abstract

Background: Arthroscopic rotator cuff repair surgery may lead to significant postoperative pain. Interscalene block (ISB) is an effective analgesic technique in these surgeries but there is a risk of the phrenic blockade. Subacromial local anesthetic infiltration is a phrenic sparing alternative technique for postoperative analgesia. The primary aim of our study was to compare the ISB with a continuous subacromial infusion (SAC) with regard to postoperative analgesia.

Methods: This prospective randomized, interventional parallel arm trial was conducted in 60 ASA grade I and II, adult patients (30 patients in each group) posted for arthroscopic rotator cuff repair surgery. Patients were randomly assigned to receive either ultrasound-guided ISB (Group ISB: 15 ml of 0.75% ropivacaine) or continuous SAC (Group SAC: 15 ml 0.75% ropivacaine as a subacromial injection by ultrasound guidance and infusion of 3 ml/hour of 0.5% ropivacaine through the catheter placed subacromial by the surgeon). Intraoperative hemodynamic parameters, visual analog scores (VAS), and rescue analgesic requirements for 24 hours, patient satisfaction, and complications were recorded.

Results: Rescue analgesic requirement was significantly higher in SAC at zero hours (P=0.000), while it was significantly higher in ISB at 12 hours (P=0.02). The VAS scores were comparable at all time points and patient satisfaction at 24 hours was similar. None of the patients had rated satisfaction related to pain relief as poor in any group. Complications like ptosis and motor weakness were seen only with ISB.

Conclusion: Both the techniques provided effective analgesia and comparable patient satisfaction with lesser incidence of complications in the SAC group. ISB provided more effective immediate postoperative pain relief while SAC was more effective in delayed analgesia for arthroscopic rotator cuff repair surgeries. SAC can be considered a reasonably safe alternative to ISB in patients with contraindications to the latter.

## Introduction

Arthroscopic rotator cuff surgery is associated with substantial pain [1]. Optimal management of such pain is necessary as it not only adds to patient disability but may delay mobilization of shoulder contributing to adhesive capsulitis [2]. This compromises the rehabilitation necessary for good functional recovery. Over the years, a variety of therapeutic modalities have been advocated for postoperative pain relief after shoulder surgery. These include regional anesthesia, intra-articular injection of local anesthetics (LA), intravenous patient-controlled analgesia (PCA) pumps, compression cooling devices, and oral analgesics [3]. Though effective, each technique is associated with some side effects.

Among regional approaches, interscalene block (ISB) is the most frequently used analgesic technique after major shoulder surgery [4]. Though, it has proven efficacy yet associated with infrequent but potentially devastating complications [5,6]. Also, good operator skill is required to achieve a successful block.

Most recently, pre-emptive LA injected into subacromial space has been shown to provide superior pain relief compared to placebo [7-9]. Analgesia has been successfully extended to the postoperative period by many authors using either disposable elastomeric or PCA pumps for continuous subacromial infusion (SAC) [10,11]. A multi-orifice catheter is accurately placed in SAC under arthroscopic guidance with minimal risk of injury to nerves, vessels, or soft tissue [12]. As analgesia delivered by pain pump is localized to shoulder region contrary to a larger portion of the extremity as in ISB, it offers the advantage of not hampering the neurological examination in the immediate postoperative period and least discomfort to patient due to motor blockade [13].

Although the efficacy of continuous SAC infusion has been substantiated previously by many authors, there are conflicting reports of the degree of pain relief and its role as an analgesic technique is not established [11,12,14]. Also, SAC has not been studied in the Indian population. We, therefore, designed to prospectively analyze and compare the analgesic efficacy of continuous SAC with single-shot ISB using ropivacaine in patients undergoing elective shoulder arthroscopy with postoperative supplemental analgesic requirement and pain control in terms of the visual analog scale (VAS) scores as the primary outcome. The incidence of any adverse effects and the patient satisfaction in terms of pain relief associated with both techniques were our secondary outcomes. We hypothesized that postoperative analgesic consumption would be lesser with ISB.

## Materials and methods

This prospective, interventional, randomized parallel-group study was conducted in the Department of Anesthesia and Critical Care at a Sports Injury Centre of a tertiary care institute of north India, after obtaining approval from the institutional ethical committee and written informed consent from all the patients. We prospectively recruited 60 adult patients of 18-60 years of age of either sex, belonging to the American Society of Anesthesiologists (ASA) grade I-II physical status, who were scheduled to undergo unilateral arthroscopic rotator cuff repair and acromioplasty under general anesthesia. Patients with any contraindications to blocks (e.g., patients with coagulopathy or on anticoagulants and infection at the local site), allergy to LA, pre-existing neurological deficit, pulmonary, cardiac, hepatic and/or renal diseases, inability to understand the pain score (VAS) and those who were morbidly obese (body mass index > 30 kg/m^2^) were excluded from the study.

Patients were randomly allocated into two groups with 30 patients in each group.

SAC (n=30): In this group patients were given 15 ml of 0.75% ropivacaine as a pre-emptive bolus in subacromial space before induction of anesthesia. In the postoperative period, ropivacaine 0.5% continuous infusion was started at 3 ml/hour for 24 hours using a disposable elastomeric pump.

ISB (n=30): In this group, patients were given ISB block before induction of anesthesia using 15 ml of 0.75% ropivacaine under ultrasound guidance.

The randomization technique used was the computerized block randomization technique.

All patients underwent a detailed pre-anesthetic checkup and were kept fasting overnight. Patients were explained about the advantages and the side effects of both techniques. The use of the VAS (0-10) was also be explained. All patients have given tablets Alprazolam 0.25 mg, Ranitidine 150 mg, and Metoclopramide 10 mg at night and two hours before surgery with a sip of water as per institutional protocol.

Patients were wheeled inside the operation room and standard monitors including non-invasive blood pressure (NIBP), electrocardiogram (ECG), pulse oximetry (SpO_2_), and neuromuscular blockade [train of four (TOF) stimulation] were attached. Oxygen was administered using a Hudson mask and an end-tidal carbon dioxide (EtCO_2_) monitor was attached; baseline heart rate (HR), mean arterial blood pressure (MAP), SpO_2__, _and respiratory rate (RR) were recorded. Intravenous access was secured using 18-20G cannula in the opposite limb and intravenous infusion of Ringer’s lactate was be started. The procedure was explained to the patients. Injection midazolam 0.02 mg/kg and injection fentanyl 1 μg/kg were administered for sedation to all the patients before performing the blocks. A single experienced anesthesiologist proficient in ultrasound-guided blocks performed all the blocks and was not involved in the study any further. Both the blocks were performed using the assistance of a high frequency (7-12 Hz) 38 mm linear ultrasound transducer (Imagic Agile, Peachtree, Georgia), after ensuring skin and probe asepsis.

Block procedure

Subacromial Catheterization Group

Patients were placed supine with the head facing away from the side to be blocked and arm displaced downward. Under all aseptic precautions, using linear ultrasound transducer (38 mm, frequency 7-12 MHz), patients were given 15 ml of 0.75% ropivacaine in subacromial space as a pre-emptive bolus dose using 20G intravenous cannula. Then, general anesthesia was administered. At the end of the surgery, a multi-orifice epidural catheter was inserted in the subacromial space by the surgeon under arthroscopic guidance. After tunneling the catheter in the subcutaneous tissue, it was connected to the elastomeric pump containing 0.5% ropivacaine which was infused at 3 ml/hour for 24 hours.

Interscalene Block

Patients were placed supine with the head facing away from the side to be blocked. Block was given using a high-frequency linear ultrasound transducer. Under all aseptic precautions, skin wheal was raised using 2% lignocaine after identification of brachial plexus between two scalene muscles. Thereafter, a 20G, 50 mm nerve block needle was advanced using a lateral to medial in-plane approach close to C5-C7 roots. After negative aspiration of blood, hydro dissection was done to exclude severe pain or resistance on injection. Then, 15 ml of 0.75% ropivacaine was injected in aliquots.

Sensory blockade was taken as a loss of sensation to alcohol sponge in C5-C7 dermatomes. Motor blockade was assessed by loss of shoulder abduction. Immediate complications such as hematoma formation, intravascular/spinal/epidural injection, Horner’s syndrome, hoarseness, or respiratory distress was assessed during this period and postoperatively.

General anesthesia was administered to all the patients using injection fentanyl 1 μg/kg and injection propofol 2-2.5 mg/kg. After checking for adequate ventilation, neuromuscular blockade was achieved with injection vecuronium 0.1 mg/kg. The trachea was then intubated with an appropriately sized endotracheal tube. All patients were mechanically ventilated. A single experienced surgeon performed all the surgeries. Anesthesia was maintained by O_2_:N_2_O (40:60) and isoflurane (0.6-1%). Injection diclofenac 75 mg intravenously was given pre-emptively as a part of multimodal analgesia. Despite the good anesthetic depth and adequate muscle relaxation, if mean arterial pressure or heart rate increases to >20% of the baseline value, patients were given a fentanyl top-up dose of 0.5-1 µg/kg assuming pain to be the cause and the need of such supplementation was noted. Intraoperative hemodynamics was also noted. Ondansetron 0.1 mg/kg intravenous was administered as prophylaxis for postoperative emesis. At the end of the surgery, muscle relaxation was reversed with injection glycopyrrolate 0.01 mg/kg and injection neostigmine 0.05 mg/kg. Patients were shifted to recovery and monitored for two hours before shifting to the ward. All patients were routinely given injection paracetamol 1 g intravenously every eight hours in the postoperative period.

Postoperative pain was evaluated at zero hours (postanesthetic care unit), 2, 6, 12, and 24 hours after surgery at rest using VAS ranging from 0 (no pain) to 10 (worst pain imaginable). Rescue analgesia was provided using injection tramadol 1 mg/kg intravenously whenever VAS was ≥4. The total amount of rescue analgesia used in 24 hours was noted. Adverse effects related to block (ptosis, dyspnoea) and catheter placement were noted. A prolonged motor blockade that was defined as the sensation of shoulder heaviness at 12 hours was noted as a complication. Patient satisfaction in terms of pain relief will be rated at 24 hours as poor, fair, good, and excellent. All the data were collected by an anesthesiologist not involved in the administration of anesthesia.

Sample size calculation was based on the mean consumption of rescue analgesia as per the study by Koltka et al. [11]. They observed that the supplemental analgesic requirements were lower in the interscalene group compared to the subacromial group (16.6% vs 53.3%, p<0.05). Taking these values as a reference, the minimum required sample size with 80% power of study and 5% level of significance is 23 patients in each study group. We decided to enroll 30 patients in each group to reduce the margin of error. So, the total sample size taken was 60.

Statistical analysis

Categorical variables are presented in number and percentage (%) and continuous variables are presented as mean ± SD and median. The normality of data was tested by the Kolmogorov-Smirnov test. Non-parametric tests were used when the normality was rejected. Quantitative variables were compared using the unpaired t-test/Mann-Whitney Test between the two groups (when the datasets were not normally distributed). The qualitative variable was compared using the Chi-Square test /Fisher’s exact test.

A p-value of <0.05 was considered statistically significant. The data were entered in MS EXCEL spreadsheet and analysis was done using Statistical Package for Social Sciences (SPSS) version 21.0 (IBM Corp., Armonk, NY).

## Results

**Figure 1:**
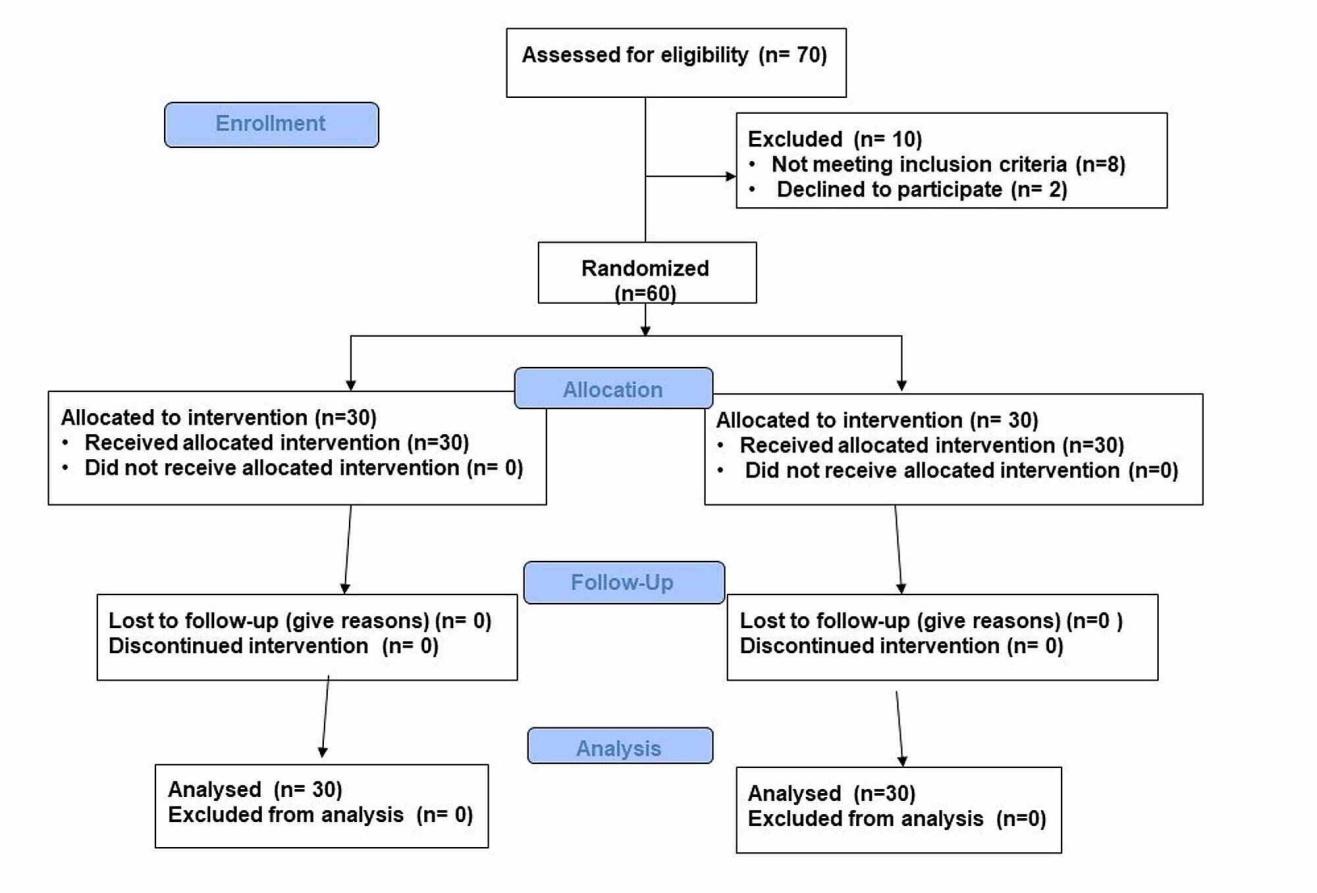
Consort flow diagram

This prospective randomized interventional study was conducted in a tertiary care center in north India. Seventy patients between 18 and 60 years of age, posted for arthroscopic rotator cuff repair and acromioplasty surgery were assessed for eligibility, and after screening for exclusion criteria, remaining 60 patients were included in the present study (Figure 1).

The demographic profile of patients was comparable in the two groups. The surgical duration, irrigation fluid volume, and neck circumference were comparable in the two groups (Table 1).

**Table 1:** Demographic parameters BMI: body mass index, SAC: subacromial infusion, ISB: interscalene block.

Parameters	Group SAC	Group ISB	P-value
Age	39.5 (9.1)	38.6 (6.7)	0.63
Weight (kg)	70.10 (11.7)	69.50 (13.2)	0.50
Height (cm)	168.3 (8.2)	169.7 (7.8)	0.85
BMI (kg/m^2^)	24.7 (3.2)	23.8 (1.9)	0.32
Neck circumference (cm)			
Preoperative	35.9 (2.4)	34.8 (1.9)	0.06
Postoperative	36.3 (2.5)	35.2 (1.9)	0.05
Irrigation fluid (Liters)	15.9 (2.4)	14.9 (2.6)	0.13
Duration of surgery (min)	54.1 (9.2)	55.7 (10.3)	0.15

Intraoperative hemodynamic parameters and oxygen saturation were comparable in the two groups. The total intraoperative fentanyl requirement was comparable (P=0.64). There was no statistically significant difference in the VAS at all time points (0, 2, 6, 12, and 24 hours). It was found that rescue analgesic requirement at zero hours was significantly higher in the SAC group (P=0.000), whereas it was significantly higher in ISB (P=0.02) at 12 hours (Table 2).

**Table 2:** Analgesic efficacy of continuous subacromial infusion compared to interscalene block VAS: visual analog scale, SAC: subacromial infusion, ISB: interscalene block.

Parameters	Group SAC	Group ISB	P-value
Intraoperative fentanyl requirement (n)	3/30	2/30	0.64
Rescue analgesia (n)			
0 hours	12/30	0/30	0.000
2 hours	9/30	4/30	0.20
6 hours	2/30	5/30	0.42
12 hours	4/30	13/30	0.02
24 hours	0/30	0/30	NS
Patient satisfaction			
Excellent	11/16/3	16/11/3	0.48
Good	16	11	
Fair	3	3	
Mean VAS scores			
0 hours	1.1 (0.9)	0.8 (0.4)	0.05
2 hours	2.3 (2.1)	2.3 (1.7)	0.44
6 hours	2.5 (2)	2.3 (2.0)	0.95
12 hours	2.5 (1.7)	3.1 (3.1)	0.06
24 hours	0.6 (0.3)	0.56 (0.40)	0.40
Complications			
Ptosis	0	2	0.24
Prolonged motor block	0	4	
Dyspnoea	0	0	
None	30	25	

The block complications like ptosis and prolonged motor blockade were observed only in the ISB group while dyspnea was not seen in any of the cases. There was no significant difference in patient satisfaction between the groups (P=0.48; Table 2). None of the patients in any group rated satisfaction in terms of analgesia as poor.

## Discussion

The results of the present study indicate that the use of both ISB and SAC provided effective analgesia and comparable patient satisfaction with lesser incidence of complications in the SAC group. ISB provided more effective immediate postoperative pain relief while SAC was more effective in delayed analgesia for arthroscopic rotator cuff repair surgeries.

Even arthroscopic shoulder surgery is known to result in intense postoperative pain which potentially interferes with the patient’s recovery and rehabilitation and may necessitate opioid therapy. The analgesic efficacy of ISB following shoulder surgeries is well proven in the literature [14,15]. Nevertheless, the occurrence of hemi-diaphragmatic palsy and several other potentially serious complications has necessitated the search for other more selective peripheral blocks [5,6]. Suprascapular nerve (SSN) provides 60-70% innervation to the shoulder joint and the rest 25-30% is by the axillary nerve (AN) [16,17]. Hence, combined blockade of the two should result in almost complete analgesia of the joint without the risk of phrenic nerve block or other serious complications. Shoulder block is the combined blockade of SSN and AN and results in effective analgesia which is comparable to ISB [18,19]. However, the need to block two separate nerves leads to increased block performance time and patient discomfort. Also, a single-shot block may not effectively cover the delayed postoperative pain. The use of continuous SAC infusion bathes the terminal endings of all the nerves supplying a shoulder joint and should result in effective analgesia. In the present study, both SAC and ISB provided effective postoperative analgesia with comparable VAS scores in the first 24 hours. These results were in agreement with that of previous researchers [8-10,20,21].

Savoie et al. compared continuous SAC of 0.25% bupivacaine at 2 ml/hour with placebo in patients undergoing arthroscopic subacromial decompression [9]. They found that the pain scores in the bupivacaine group were significantly lower as compared to the control group. Cho et al. [20] and Park et al. [21] also documented good pain relief with the use of 0.5% SACs in their studies. There had been concerns of glenohumeral chondrolysis with bupivacaine infiltration inside the joint though these claims have not been substantiated clinically as well as radiologically at the end of one year in a study by Busfield et al. [12]. Ropivacaine seems to have a safer profile, and hence, it was used for initial injection and subsequent infusion [22]. Harvey et al. [8] evaluated the efficacy of subacromial PCA infusion of 0.2% ropivacaine versus saline for postoperative pain control following arthroscopic shoulder surgery. The use of a PCA ropivacaine infusion provided effective postoperative pain control as depicted by a significant reduction of postoperative pain by 34% as measured by VAS.

Webb et al. [10] evaluated the analgesic efficacy and complication rate of a single-shot ISB with a continuous SAC of 0.5% bupivacaine. They found no difference between ISB versus continuous SAC of a LA regarding efficacy, rescue medication intake, and complication rate. Their findings coincide with these study findings of similar VAS scores and comparable rescue analgesic requirements and minimal complications.

Early pain relief in terms of rescue analgesic requirement and VAS was superior in the ISB group. This can be expected as proximal brachial plexus blockade by ISB provides more extensive and effective coverage of shoulder joint and provides excellent early postoperative analgesia. The early pain can be due to overdistension of the joint capsule caused by irrigation fluid used intraoperatively, as it takes up to 12 hours to get absorbed [18]. Incomplete and less dense coverage of shoulder innervation resulted in some pain in the SAC group. It is also possible that there was a dilution of the LA infused by the irrigation fluid in the early postoperative period. Previous researchers have also reported superior pain control in the early postoperative period with ISB [11,14].

Koltka et al. [11] compared the efficacy of postoperative subacromial and interscalene continuous infusion following ISB using levobupivacaine in 60 patients. They found that VAS scores were <4 at most time points in both the groups; but they were significantly lower in the interscalene group. Additional analgesic requirements were lower in the interscalene group (16.6% vs 53.3%). One patient had toxicity related to ISB, but there was no complication related to subacromial catheters. Patients’ satisfaction was higher in the interscalene group. This study demonstrated that SACs provided good postoperative analgesia but are not as effective as interscalene infusion. The relatively similar pain control found in our study in the two groups is likely because we did not provide continuous interscalene infusion, and hence, delayed pain relief was better in the SAC group. Also, the use of pre-emptive ultrasound-guided subacromial injection and the use of multi-modal analgesia improved the quality of pain relief. Rescue analgesia requirement at 12 hours was significantly lesser in the SAC group (P=0.02). This can be explained by the rebound pain phenomenon with single-shot ISB when it wears off and this has been reported by many authors previously [18,23]. A meta-analysis conducted by Abdallah et al. had concluded that administration of single-shot ISB can generally provide effective analgesia only up to 8 hours following shoulder surgery with no discernible advantages afterward [24].

Ptosis was seen in two patients and prolonged motor block was seen in four patients in the ISB group while no complications were recorded in the SAC group. Ptosis is a common complication of ISB as a component of Horner’s syndrome and results from the diffusion of LA paravertebrally to block the cervical sympathetic chain. Extensive motor blockade of the upper limb is a significant cause of discomfort to most patients following ISB, despite its excellent quality of analgesia. Dhir et al. also reported significantly higher motor weakness in the ISB group as compared to a peripheral block (ShB) [18]. We did not find the complication of dyspnoea consequent to phrenic nerve block in any of our patients. This may be because diaphragmatic palsy was assessed only clinically by subjective complaints of dyspnoea and not objectively demonstrated using ultrasonography and spirometry. However, phrenic nerve blockade is expected to occur in most patients with the LA volume used in the current study for ISB while SAC is known to be a diaphragm sparing block [14,15]. Previous trials have also reported higher complications with ISB as compared to SAC [13,14].

Limitations

There are certain limitations to our study. First, the blocks were performed by an experienced operator, so the results may not be the same in hands of inexperienced operators. Second, patient and outcome assessor blinding was not feasible because of the different sites of injection and the use of catheters with continuous infusions in the SAC group. However, the investigators assessed the outcomes without being involved in the block procedure to minimize any possibility of bias. Lastly, VAS scores were assessed only at rest and dynamic scores could not be assessed because of the application of shoulder brace by the surgeons, prohibiting any movement on the shoulder joint.

## Conclusions

SAC is equally efficacious as ISB for arthroscopic shoulder surgeries in terms of intraoperative fentanyl consumption, postoperative VAS scores recorded over the first 24 hours, and patient satisfaction regarding the quality of analgesia. Side effects and complications like ptosis and motor weakness were seen exclusively in the ISB group. ISB provided more effective immediate postoperative pain relief while SAC was more effective in delayed analgesia for arthroscopic rotator cuff repair surgeries. ISB is known to lead to phrenic nerve blockade with commonly used dosages which can be detrimental to patients with lung pathology. SAC blocks the terminal endings of nerves supplying the shoulder joint and is, therefore, a phrenic sparing block. Hence, continuous SAC of LA can be considered a reasonable alternative to ISB in patients with contraindications to later. Large multicentric studies comparing both the blocks are desirable in the future to prove this conclusively.
